# The Story of the Insane from Year to Year

**Published:** 1900-05-19

**Authors:** 


### THE STORY OF THE INSANE FROM YEAR TO YEAR.

Twelfth Series.

### THE MIDLOTHIAN ASYLUM AT ROSSLYNLEE, EDINBURGH.

The report comes to us without the usual statistical tables. At the beginning of the year it contained 229 patients, and at the end of it there were 234 in residence, and this was 71 in excess of the number for which space is provided. There were 17 private patients in residence. There were 80 admissions, 48 discharges, and 26 deaths. Twenty-four of the cases admitted owed their insanity to hereditary tendency and 15 to drink. The recoveries stand at 42·5 per cent. of the admissions, and the deaths at 11·3 on the average number resident. Seven of the deaths were caused by brain diseases, 3 by consumption, 1 by pleurisy, and 1 by pneumonia. Dr. Mitchell has had a great many changes in his staff during 1898, for not fewer than 21 have either left voluntarily or have been dismissed. It is a fact that in all institutions the staff will occasionally become unsettled without any apparent reason, and after one of these periods of unstable equilibrium it goes on smoothly for a long time again without untoward event. At present £65,000 are being spent on additions to the asylum. These comprise hospital blocks for men and women, observation blocks, cottages, engine house, &c

### THE LANARK DISTRICT ASYLUM AT HARTWOOD.

At the end of the year there were 570 patients in residence. The first admissions were 154, the readmissions were 102, the recoveries 93, and the deaths 58. Of these deaths 15 were caused by cerebral and spinal diseases, 9 by consumption and other tuberculous diseases, 1 by conjestion of the lungs, and 5 by pneumonia. Of the admissions 22 owed their insanity to moral causes, 44 to intemperance in drink, 20 to hereditary influence, and in 33 the cause was unknown. The recoveries were at the rate of 36·3 per cent. of the admissions, and the deaths were 11 per cent. of the average number resident. It is very rarely that we have to chronicle such enlightened action on the part of a committee as we have here, for additions have been made to raise the total number of beds to 950. As Dr. Clark truly remarks, the committee "have established a wise precedent, for in this way only can facilities for early treatment of insanity be always secured." Not only that, but the patients have not to be sent to other counties for treatment, and thus money is saved to the district, and the vacant accommodation is used as a source of income. This has been done at Lanark. We are told that private patients are received at a lower rate than at any asylum in Scotland, and we can readily understand that these low rates of board have proved a boon to many a family. In many of our lunatic hospitals in England which are supposed to be "charitable" the patients pay more than they do in these district and Royal asylums in Scotland where "charity" is not talked about at all. In the treatment of insanity by spleen extract Dr. Clark gives us the benefit of a more extended experience, especially in those cases where other treatment has failed; and, beyond doubt, that experience is encouraging. Nursing classes are held in this asylum, and seven of the staff went up for the examination of the Medico-Psychological Association, and all the seven passed. The committee say "that the management of the asylum continues to reflect the greatest credit upon Dr. Campbell Clark and those associated with him." The weekly rate of maintenance is 8s. 9d. a head for the district. In the next report we should like to see a scale of the charges for private patients and the number received at the various charges.

